# Hemoglobin Lepore‐Boston‐Washington: A Rare Cause of Unmeasurable HbA1c and Diagnostic Challenge in Diabetes

**DOI:** 10.1002/jcla.70325

**Published:** 2026-07-29

**Authors:** Filippo Russo, Silvia Trombetti, Maria Rosaria Storino, Raffaele Sessa, Carmela De Felice, Giuseppe Schiavone, Michela Grosso

**Affiliations:** ^1^ Department of Molecular Medicine and Medical Biotechnology University of Naples Federico II Naples Italy; ^2^ CEINGE‐Biotecnologie Avanzate F. Salvatore Naples Italy; ^3^ AORN “A. Cardarelli” UOC Patologia Clinica Naples Italy; ^4^ Department of Laboratory Medicine AOU Federico II Naples Italy

To the Editor,

Hemoglobin Lepore variants comprise a small group of structurally abnormal hemoglobins with a thalassemia‐like phenotype. They result from the in‐frame fusion of the 5′ end of the δ‐globin gene (HBD) with the 3′ end of the *β*‐globin gene (HBB) and have been identified across diverse ethnic groups [1]. When co‐inherited with β‐thalassemic alleles, Hb Lepore produces a wide clinical spectrum, from non‐transfusion‐dependent to transfusion‐dependent thalassemia, depending on the degree of globin chain imbalance and compensatory HbF production. Hb Lepore‐Boston‐Washington is the most prevalent Lepore variant (HbVar database: https://globin.cse.psu.edu/). It is relatively common in Southern Italy, particularly in the Campania region, where its prevalence has been recently characterized by Ricchi et al. [2]. The fusion gene (δ87/β116) consists of the 5′ portion of the *δ*‐gene (from the 5′ UTR to codon 87 of exon 2) fused to the 3′ portion of the *β*‐gene (from intron 2 nucleotide +8 to the 3′ UTR). The two genes share a 58‐base‐pair region of homology at the crossover junction [3]. In heterozygous carriers, the presence of a functional *β*‐globin allele allows for the formation of adult hemoglobin (HbA_0_), thereby ensuring the analytical feasibility of HbA1c measurement. Homozygosity for Hb Lepore‐Boston‐Washington is a very rare condition in which β‐globin synthesis is virtually abolished, leading to an absence of HbA_0_ [1].

Diabetes Mellitus (DM) is a chronic metabolic disorder resulting from defects in insulin secretion, insulin action, or both [4, 5]. According to the International Diabetes Federation (IDF), approximately 66 million adults in Europe are living with diabetes, emphasizing the critical need for accurate diagnostic and monitoring strategies [6, 7]. In particular, Type 1 Diabetes Mellitus (T1D) accounts for approximately 5%–10% of all diabetes cases and is caused by autoimmune destruction of pancreatic β‐cells, leading to absolute insulin deficiency [8]. Chronic hyperglycemia is associated with long‐term vascular complications, including diabetic retinopathy, nephropathy, neuropathy, and increased cardiovascular risk [9]. Hemoglobin A1c (HbA1c) remains the gold standard for assessing glycemic status in diabetic patients, serving as a critical metric to guide therapeutic decisions and long‐term clinical management [10]. HbA1c originates through the spontaneous, non‐enzymatic glycation of the N‐terminal valine residue on the β‐chain of normal adult hemoglobin (HbA_0_) [11]. Due to the approximately 120‐day lifespan of erythrocytes, HbA1c levels reflect average blood glucose concentrations over the preceding 8–12 weeks. Since 2010, the American Diabetes Association (ADA) has recognized HbA1c ≥ 6.5% as a diagnostic criterion for diabetes mellitus, further cementing its clinical importance [12]. The most widely used method for HbA1c quantification is cation‐exchange high‐performance liquid chromatography (CE‐HPLC) or capillary electrophoresis (CE), which separates hemoglobin fractions based on differences in charge, molecular weight, and polarity [13].

Herein, we present a rare and instructive case of a T1D patient homozygous for hemoglobin Lepore‐Boston‐Washington, in whom HbA1c was undetectable due to the complete absence of HbA_0_. This case underscores the critical importance of recognizing hemoglobinopathy‐related interference, the value of molecular diagnostics, and the need for alternative glycemic biomarkers to ensure personalized diabetes management.

A 49‐year‐old Caucasian woman from the Campania region (Southern Italy) was referred to the Diabetes Unit of the Azienda Ospedaliera Universitaria Federico II (Naples) in June 2022 for a routine follow‐up. The patient, with a documented history of type 1 diabetes (T1D), presented for routine glycemic monitoring in the absence of any acute clinical signs or complications. She had previously undergone a splenectomy and had a history of sporadic red blood cell transfusions. Diabetes management consisted of insulin therapy combined with an oral hypoglycemic agent; her body mass index was within the normal range. Continuous self‐monitoring blood glucose data were not available. HbA1c was initially analyzed on a Variant II HPLC (CE‐HPLC) (Bio‐Rad Laboratories, Hercules, CA, USA) with the HbA1c kit (Figure 1A) and subsequently with the HbA_2_/HbA1c Dual kit (Variant II, Bio‐Rad Laboratories) (Figure 1B). Both assays failed to detect the HbA1c and HbA_0_ peaks within the expected analytical retention time (RT) windows. In addition, the Variant II HbA_2_/HbA1c Dual Program revealed an abnormal hemoglobin fraction (Hb X = 10.3%, RT = 2.737) co‐eluting with the HbA_2_ fraction—consistent with an HbA_2_/Hb Lepore fraction—together with a markedly elevated HbF level (75%; normal adult range: 0%–1%). Results were further validated by capillary zone electrophoresis (CE) analysis (Sebia Capillarys 2 Flex Piercing System, Sebia, Lisses, France), which confirmed elevated HbF levels (88.8%), the presence of an abnormal peak (9.8%), and the absence of detectable HbA_0_ and HbA1c fractions (Figure 1C). Noteworthy, a quantitative discrepancy in HbF levels was observed between the two analytical methods, yielding 75.0% by CE‐HPLC (Dual Kit) and 88.8% by Capillary Electrophoresis (CE). This variation is a known phenomenon in patients with extremely high fetal hemoglobin levels or complex hemoglobinopathies. In CE‐HPLC, the presence of massive abnormal fractions and unidentified peaks, such as those detected at RT 0.57 and 1.73 min with the HbA1c kit, can alter column chromatography dynamics, leading to inaccurate peak area integration and an underestimation of the true HbF percentage. Conversely, CE provides a superior resolution based on the charge‐to‐mass ratio, avoiding the integration artifacts typical of HPLC in the presence of rare variants [14, 15]. Therefore, the CE measurement may provide a more accurate estimate of the patient's HbF level. Furthermore, unlike CE‐HPLC, where Hb Lepore co‐elutes within the HbA_2_ window preventing its isolation, CE successfully separates and quantifies Hb Lepore as a distinct peak (HbX = 9.8%).

**FIGURE 1:**
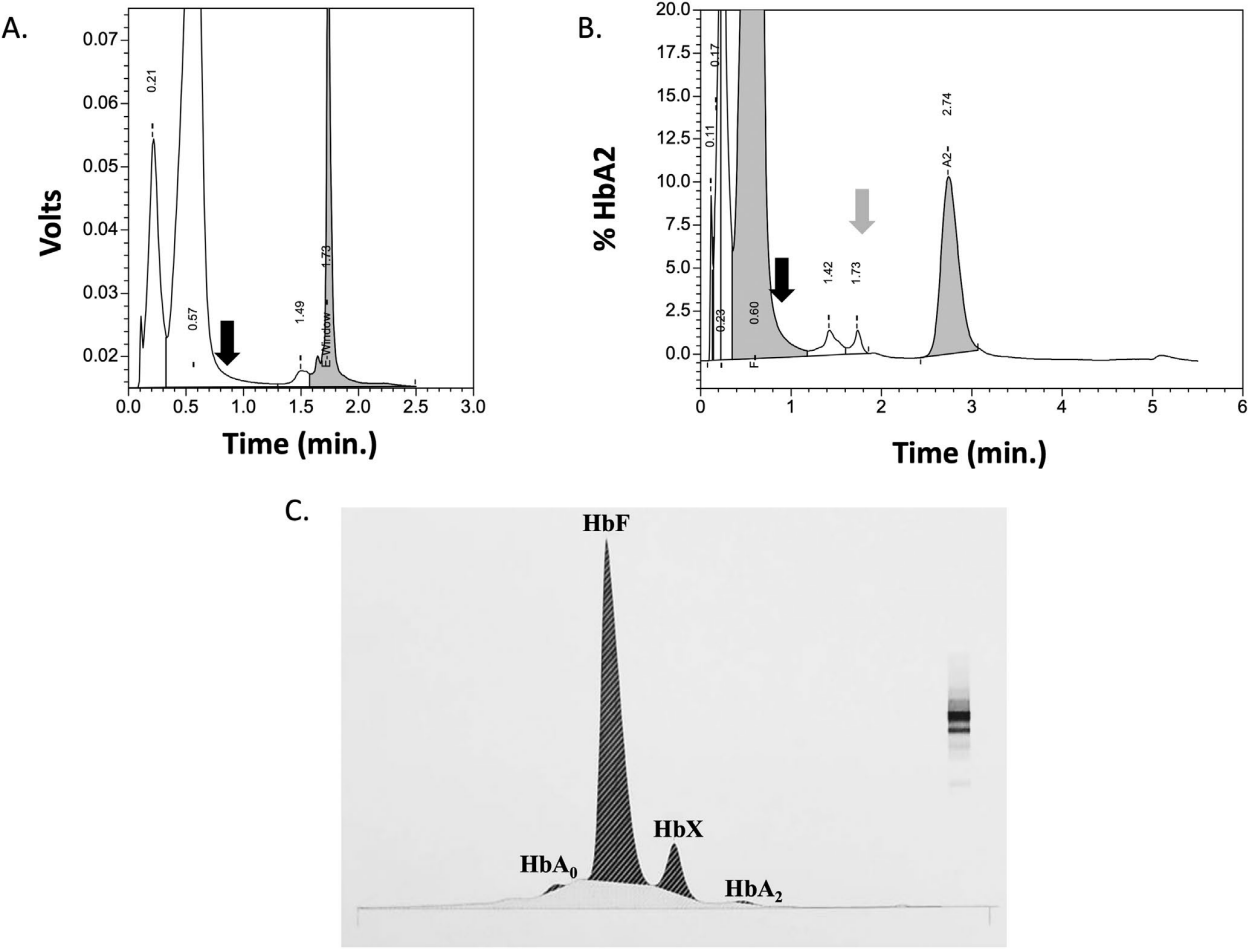
Combined analytical evaluation of the hemoglobin profile of the patient. (A) Cation‐exchange high‐performance liquid chromatography (CE‐HPLC) elution profile obtained with the Variant II HbA1c kit (Bio‐Rad Laboratories), revealing the absence of the HbA1c peak (black arrow) and the presence of two unidentified hemoglobin fractions at retention times (RT) of 0.57 min (67.8%) and 1.73 min (14.3%). (B) CE‐HPLC elution profile obtained with the Variant II Dual kit (Bio‐Rad Laboratories), showing the absence of both HbA1c (black arrow) and HbA0 (grey arrow); the minor peak in this window (0.6%) is considered an analytical artefact. A prominent fraction of fetal hemoglobin (HbF = 75.0%, RT 0.60 min) and an abnormal peak in the HbA2 window (HbA2/Hb Lepore fraction; 10.3%, RT 2.74 min) are clearly visible. The x‐axis represents the RT (min), and the y‐axis represents the detector signal intensity, expressed in Volts for panel A and as a relative percentage/absorbance for panel B. (C) Capillary electrophoresis (CE) electropherogram (Sebia Capillarys 2 Flex Piercing System) confirming the atypical hemoglobin profile. Although a quantitative divergence in the massive HbF fraction is observed compared to CE‐HPLC (HbF = 88.8% and an unidentified HbX fraction = 9.8%), CE rules out chromatographic integration artifacts and confirms the near‐total absence of adult hemoglobins.

For further laboratory evaluation, patient's blood samples were collected in EDTA‐ and serum‐separator tubes. Hematological parameters were determined on an automated cell counter (ADVIA 2120 I, Siemens, Munich, Germany) (Table 1). Results revealed a moderate anemia (Hb = 87 g/L) with microcytic hypochromic red cell indices typical of thalassemia (Table 1). Biochemical analyses were performed with an automated clinical chemistry analyzer (Architect c16000, Abbott Diagnostics, Abbott Park, IL, USA). Serum iron levels (17.0 μmol/L) were within the normal reference range (9.0–30.4 μmol/L). Conversely, total bilirubin levels were significantly elevated (46.2 μmol/L; reference range: 3.4–20.5 μmol/L). In January 2023, the patient was re‐evaluated and results confirmed the previous laboratory findings (Table 1). During the follow‐up, serum fructosamine was measured as an alternative marker of glycation using a colorimetric assay (Architect c16000) revealing elevated fructosamine levels (367 μmol/L, reference range: 205–285 μmol/L). The elevated fructosamine value coincided with a normal fasting plasma glucose level at the same evaluation (5.4 mmol/L; reference range: 3.9–6.1 mmol/L). This discrepancy indicates sub‐optimal glycemic control over the preceding 2–3 weeks, underscoring the need for therapeutic adjustment in this patient. Renal function was preserved with a urea level of 6.3 mmol/L (reference range 3.5–7.2 mmol/L) and an eGFR [CKD‐EPI] of 111 mL/min/1.73 m^2^ (reference range > 90 mL/min/1.73 m^2^). Concurrently, serum albumin (42 g/L, reference range: 35–52 g/L), total proteins (76 g/L, reference range: 64–83 g/L) and C‐reactive protein (1.9 mg/L, reference range: 0–5.0 mg/L) were all within normal limits.

**TABLE 1:** Hematological and biochemical data at first and follow‐up evaluation.

Test	Unit	Reference range	June 2022	January 2023
RBC	×10^12^/L	4.0–5.0	3.8	4.1
Hb	g/L	120–155	87	94
HCT	%	35.0–48.0	28.5	29.8
MCV	fL	80.0–97.0	74.3	71.9
MCH	pg	25.0–34.0	22.7	22.7
MCHC	g/L	320–380	306	316
RDW	%	11.0–15.0	23.3	22.3
PLT	x10^9^/L	150–450	852	955
PCT	%	—	0.9	0.8
MPV	fL	7.1–10.0	10.6	8.3
PDW	%	—	43.8	63.3
WBC	x10^9^/L	4.5–11.0	21.4	11.8
Neutrophils (%)	%	40–70	22.0	46.0
Neutrophils (#)	x10^9^/L	1.8–7.0	4.7	5.4
Lymphocytes (%)	%	20–45	69.0	40.9
Lymphocytes (#)	x10^9^/L	1.0–4.8	14.7	4.8
Monocytes (%)	%	—	4.6	9.6
Monocytes (#)	x10^9^/L	0.1–0.8	0.9	1.1
Eosinophils (%)	%	0–6	1.1	2.0
Eosinophils (#)	x10^9^/L	0.0–0.45	0.2	0.2
Basophils (%)	%	0–1.5	0.4	1.5
Basophils (#)	x10^9^/L	0.0–0.20	0.1	0.2
Total Bilirubin	μmol/L	3.4–20.5	46.2	29.1
Direct Bilirubin	μmol/L	0–8.6	12.0	12.0
Glucose	mmol/L	3.9–6.1	2.3	5.4
Fructosamine	μmol/L	205–285	—	367
Serum Iron	μmol/L	9.0–30.4	17.0	20.0
Ferritin	μg/L	5–204	—	537
HPLC—HbF window	%	0.0–1.1	75.0	74.9
HPLC—HbA2 window	%	2.5–3.2	10.3	11.5
CE‐HbF window	%	0–5	—	88.8
CE‐ X window	%	—	—	9.8

*Note:* The abnormal fraction co‐eluting within the HbA2 window on cation‐exchange HPLC likely represents Hb Lepore, potentially co‐existing with true HbA2.

Abbreviations: HbA_2_, hemoglobin A_2_ fraction; HbF, fetal hemoglobin; Hb, hemoglobin; HCT, hematocrit; MCV, mean corpuscular volume; MCH, mean corpuscular hemoglobin; MCHC, mean corpuscular hemoglobin concentration; MPV, mean platelet volume; PCT, plateletcrit; PDW, platelet distribution width; PLT, platelet count; RBC, red blood cell count; RDW, red cell distribution width; WBC, white blood cell count.

At the initial evaluation, serum iron alone was insufficient to exclude iron deficiency. A complete iron panel obtained at re‐evaluation revealed markedly elevated ferritin (537 μg/L; reference range: 5–204 μg/L) and a high transferrin saturation (approximately 78%). Concurrently, a normal C‐reactive protein level (1.9 mg/L) indicated that the hyperferritinemia was not attributable to an acute‐phase response. The soluble transferrin receptor was also elevated (7.25 mg/L); in the presence of high ferritin and transferrin saturation, this is best interpreted as a marker of expanded, ineffective erythropoiesis rather than iron deficiency. Taken together with the hyperbilirubinemia, these findings excluded iron deficiency and were consistent with iron overload secondary to chronic hemolysis, ineffective erythropoiesis, and the patient's transfusion history. Consequently, the microcytic, hypochromic red cell indices were attributable to the underlying hemoglobinopathy. Following best practice guidelines for hemoglobinopathies, further investigation was undertaken to clarify the diagnosis [16]. Given the anomalous hemoglobin HPLC elution profile, Multiplex Ligation‐dependent Probe Amplification (MLPA) was performed to evaluate the presence of large rearrangements in the HBB cluster with a SALSA MLPA Probemix P102‐D1 HBB kit (MRC‐Holland, Amsterdam, Netherlands) as previously described [17]. MLPA analysis revealed absence of signals corresponding to probes from the 3′ end of the HBD gene (*δ*‐globin, exon 3 position +399 nt) to the 5′ end of the HBB gene (β‐globin, intron 1 position +154 nt). As the observed MLPA pattern was suggestive of homozygous Hb Lepore‐Boston‐Washington (Figure 2A), GAP‐PCR was performed, confirming the diagnosis (Figure 2B,C) [18, 19].

**FIGURE 2:**
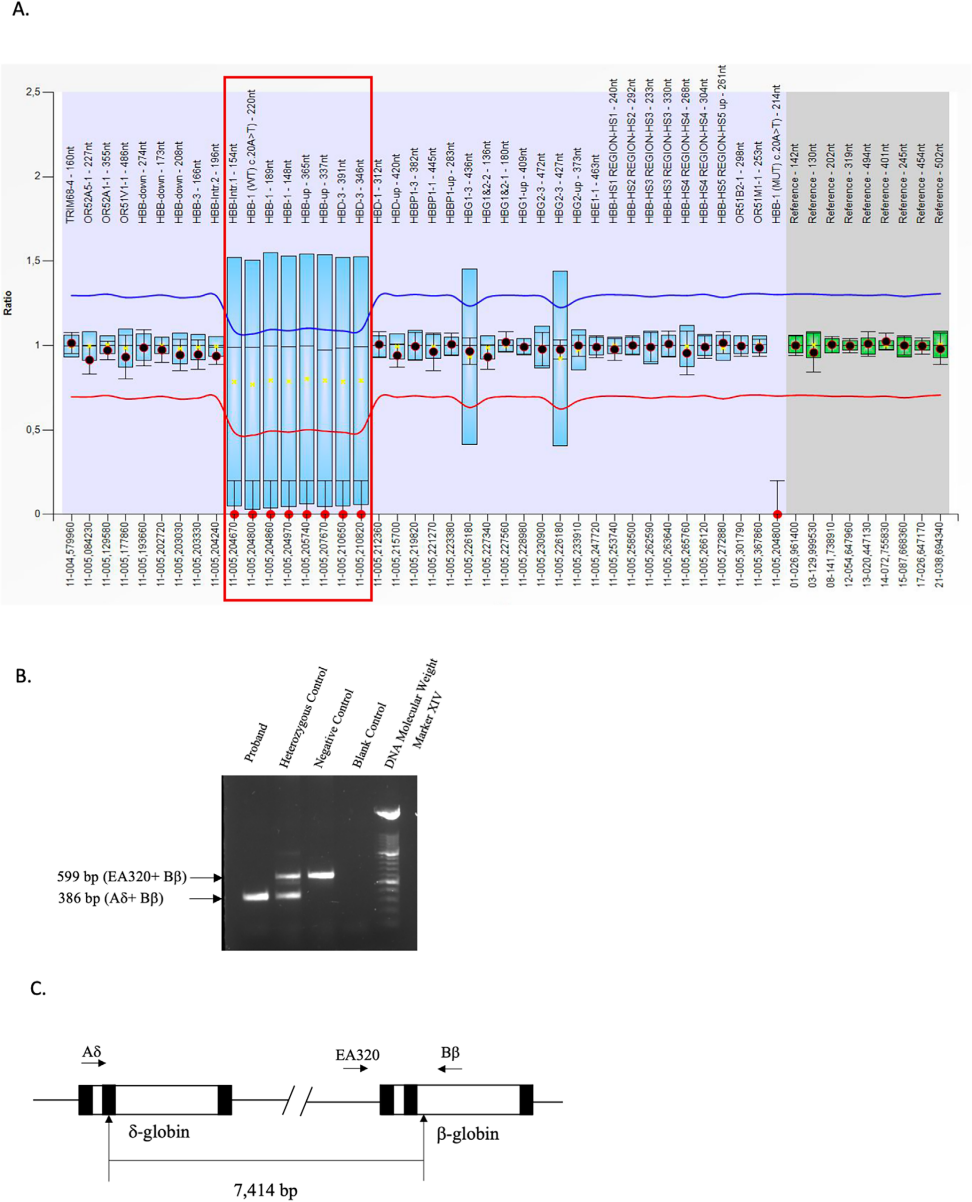
Molecular characterization of the Hb Lepore‐Boston‐Washington deletion. (A) Multiplex ligation‐dependent probe amplification (MLPA) profile obtained using the SALSA MLPA Kit P102 HBB (lot D1‐0920; MRC Holland, Amsterdam, The Netherlands). The horizontal axis displays the MLPA probes arranged according to their chromosomal coordinates, while the vertical axis shows the normalized probe ratio. Probes falling below the deletion cut‐off (0.70 to 0.5) indicate a gene deletion (red spots). The complete absence of signals from probe HBD‐3‐346 nt to probe HBB‐intr1‐154 nt is consistent with a homozygous Hb Lepore‐Boston‐Washington deletion. Red rectangle indicates all the deleted probes. (B) GAP‐PCR analysis of the Hb Lepore‐Boston‐Washington variant resolved on a 1.0% agarose gel. Lane 1: Proband (homozygous); Lane 2: Heterozygous control; Lane 3: Negative control; Lane 4: Blank control; Lane 5: DNA Molecular Weight Marker XIV (100 bp ladder, Cat. No. 11721933001; Roche Diagnostics). The amplification pattern confirms the homozygous state of the patient. (C) Schematic representation of the primer binding sites used in the GAP‐PCR and mapping of the specific breakpoints for the Hb Lepore‐Boston‐Washington deletion (7414 bp) within the β‐globin gene cluster.

This molecular characterization accounted for the abnormal HPLC profile and the subsequent inability to measure HbA1c. Consequently, glycemic monitoring was transitioned to fructosamine, as it was readily available within the local laboratory workflow [20]. Glycated albumin represents another valid alternative, offering similar temporal coverage and independence from red blood cell survival. Both markers reflect the glycation of circulating serum proteins over the preceding 2–4 weeks, are unaffected by hemoglobin variants, and can be measured on standard chemistry platforms [21]. In this patient, elevated fructosamine levels suggested suboptimal glycemic control, offering a clinically actionable surrogate in the absence of measurable HbA1c, though it should be noted that both markers can be influenced by body mass index and disorders of protein metabolism [22]. In our patient, however, these potential confounders were absent: renal function was preserved, serum albumin and total proteins were normal, there was no biochemical evidence of inflammation, and the body mass index was within the normal range. Consequently, the reliability of fructosamine as a surrogate marker of glycemic control was not compromised in this clinical context. Conversely, fructosamine possesses intrinsic limitations that warrant consideration in this specific case. It reflects glycemic control over a narrower window than HbA1c and depends heavily on serum protein concentration and turnover. Moreover, it is less standardized than HbA1c and lacks specific validation in patients with hemoglobinopathies. Finally, continuous glucose monitoring (CGM) and self‐monitoring of blood glucose (SMBG) data were unavailable; the absence of these longitudinal metrics represents a key limitation of the present report. Another noteworthy clinical finding in this patient was the markedly elevated HbF level (75%) which likely mitigated the severity of the anemia (Hb 87 g/L) by partially compensating for the lack of HbA_0_. We hypothesize that the homozygous Hb Lepore defect, by eliminating the adult β‐promoter, may force the Locus Control Region (LCR) to persistently activate the upstream γ‐globin genes [23]. This transcriptional redirection, compounded by the intense erythropoietic stress arising from unpaired α‐chains, could effectively bypass physiological HbF silencing, and might account for both the massive HbF production and the chronic hemolytic profile observed in our patient.

In conclusion, this case illustrates a rare and instructive clinical condition in which homozygosity for Hb Lepore‐Boston‐Washington precluded HbA1c‐based diabetes monitoring. The absence of HbA_0_ explained the analytical failure of the HbA1c assay, highlighting the risk of relying uncritically on this parameter in patients with unrecognized hemoglobinopathies. In this setting, alternative glycemic markers—such as fructosamine—allowed for a consistent assessment of glycemic control and informed therapeutic decisions. Simultaneously, targeted molecular diagnostics proved essential to elucidate the underlying hemoglobin defect and correlate the abnormal hemoglobin profile with the patient's genotype. Overall, this case underscores the need for close collaboration between clinicians and laboratory specialists, and the importance of individualized monitoring strategies whenever rare hemoglobin variants interfere with standard biomarkers.

## Funding

The authors have nothing to report.

## Ethics Statement

The study was conducted according to the guidelines of the Declaration of Helsinki and approved by the Institutional Ethics Committee of University of Naples Federico II (protocol code 443/21; date of approval: 24/02/2022).

## Consent

The patient provided written informed consent to participate in the study and to the use of her biological samples for research purposes.

## Conflicts of Interest

The authors declare no conflicts of interest.

## Data Availability

The data that support the findings of this study are available from the corresponding author upon reasonable request.
